# Performance of a new single-use bronchoscope versus a marketed single-use comparator: a bench study

**DOI:** 10.1186/s12890-022-01982-4

**Published:** 2022-05-12

**Authors:** Carla R. Lamb, Ekaterina Yavarovich, Vincent Kang, Elliot L. Servais, Lori B. Sheehan, Sara Shadchehr, James Weldon, Matthew J. Rousseau, Gregory P. Tirrell

**Affiliations:** 1 Division of Pulmonary and Critical Care, Lahey Hospital and Medical Center, 41 Burlington Mall Road, Burlington, MA 01805 USA; 2 Division of Thoracic Surgery, Lahey Hospital and Medical Center, Burlington, MA USA; 3 Division of Anesthesiology, Lahey Hospital and Medical Center, Burlington, MA USA; 4 Department of Medicine, Tufts Medical Center, Boston, USA; 5 Boston Scientific Corporation, Marlborough, MA USA

**Keywords:** Bronchoscopy, Disposable equipment, Interventional pulmonology, Medical technology

## Abstract

**Background:**

Single-use flexible bronchoscopes eliminate cross contamination from reusable bronchoscopes and are cost-effective in a number of clinical settings. The present bench study aimed to compare the performance of a new single-use bronchoscope (Boston Scientific EXALT Model B) to a marketed single-use comparator (Ambu aScope 4), each in slim, regular and large diameters.

**Methods:**

Three bronchoscopy tasks were performed: water suction and visualization, “mucus” mass (synthetic mucoid mixture) suctioned in 30 s, and “mucus” plug (thicker mucoid mixture) suction. Suction ability, task completion times, and subjective ratings of visualization and overall performance on a scale of one to 10 (best) were compared. All bronchoscopy tasks were completed by 15 physicians representing diversity in specialization including pulmonary, interventional pulmonary, critical care, anesthesia, and thoracic surgery. Each physician utilized the six bronchoscope versions with block randomization by bronchoscope and task.

**Results:**

Aspirated mean mass of “mucus” using EXALT Model B Regular was comparable to that for an aScope 4 Large (41.8 ± 8.3 g vs. 41.5 ± 5.7 g respectively, *p* = 0.914). In comparisons of scopes with the same outer diameter, the aspirated mean mass by weight of water and “mucus” was significantly greater for EXALT Model B than for aScope 4 (*p* < 0.001 for all three diameters). Mean ratings for visualization attributes were significantly better for EXALT Model B compared to aScope 4 (*p*-value range 0.001−0.029).

**Conclusion:**

A new single-use bronchoscope provided strong suction capability and visualization compared to same-diameter marketed single-use comparators in a bench model simulation.

**Supplementary Information:**

The online version contains supplementary material available at 10.1186/s12890-022-01982-4.

## Background

Bronchoscopy remains a cornerstone diagnostic aid to identify the etiology of pulmonary radiographic abnormalities [1]. It has diagnostic uses in infections, neoplasms, hemoptysis, and therapeutic indications including clearance of airway secretions, mucus plugging and relief of airway obstructions, airway management (i.e., intubation and endobronchial blocker placement, and airway evaluation during pulmonary surgery), foreign body retrieval, percutaneous tracheostomy and balloon dilatation with stent placement for tracheobronchial stenosis [2–6].

A 2020 systematic review and cost-effectiveness analysis including 16 studies reported benefits of single-use compared to reusable flexible bronchoscopes in terms of cost effectiveness, cross-contamination and resource utilization, with cross-contamination or infection rates of 2.8% vs. 0% reported for reusable vs. single-use bronchoscopes in this analysis, respectively [7]. Based on consensus opinion, the American Association for Bronchology and Interventional Pulmonology recommended that “disposable bronchoscopes should be used first line when available” if bronchoscopy is needed in patients with suspected or confirmed COVID-19 infection [8]. At least three brands of single-use bronchoscopes have been developed and studied, commonly in anesthesia settings [9]. In a 2020 cross-sectional user satisfaction study in 21 Spanish pulmonology services, the newest model (4th generation) of the most widely used single-use bronchoscope received high ratings for ease of use, imaging and suction [10]. Other studies have focused on advantages or disadvantages of specific features of these devices, such as size and suction capacity. For example, in a simulation study using a manikin, a slim model of a single-use bronchoscope required more time for nasal intubation than a conventional reusable bronchoscope, and was assessed as requiring more rigidity (to be comparable to the reusable) for management of a difficult airway [11]. A 2014 study of a single-use bronchoscope in an animal experiment and later in three intensive care units (ICU) reported that a large working channel can be advantageous if adequate suction capability is demonstrated [12]. In a study of 10 healthy volunteers, bronchoscopists achieved greater bronchoalveolar lavage (BAL) aspirated volume using a single-use flexible bronchoscope compared to a conventional bronchoscope (152 ml vs. 124 ml respectively, *p* ≤ 0.010), with no significant difference between the cell yield and viability between the methods [13].

In the current study, we systematically evaluate a new single-use flexible bronchoscope developed for endoscopic procedures within the airways and tracheobronchial tree. The aim of the study was for procedural experts to assess performance of this new device compared to a marketed single-use bronchoscope on three standard bronchoscopy tasks in a preclinical protocol-guided bench study.

## Materials and methods

Because this study was a bench simulation that did not include observation, interaction with, or intervention with living individuals to gather information, approval from research ethics committees and institutional review boards was not required. All methods were carried out in accordance with guidelines and regulations for research conduct in the Center for Professional Development and Simulation at Lahey Hospital & Medical Center. The physician investigators shown in the images in this manuscript provided informed consent to be photographed or recorded in videos during the bench simulation, and for publication of the images.

### Comparison of bronchoscopes in a bronchoscopy simulation study

The “Airway Larry” Airway Management Trainer (Nasco Healthcare, Saugerties, New York, USA) pulmonary bench model was used to simulate an adult patient for practicing suction techniques. The model includes realistic anatomy and landmarks to allow practice of oral, digital, and nasal intubation and insertion of tubes for airway management. Distinct landmarks of the model include the carina and the left and right main airway. The model was modified to meet the needs of this study; for example, a Custom Biliary Tract Model (Pulse Medical Demonstration Models, Holland, Pennsylvania, USA) was used to test visualization in the left main airway of the model after it was determined to fairly represent possible colors of the respiratory mucosa. An 8.0 mm endotracheal tube was used for “intubation” of the model during the study. Other materials used in the bench simulation were a “mucus” fluid media (homogenous mixture of water and 1% guar gum by volume to simulate “mucus”, water and 3% guar gum to simulate “mucus” plug) and a mucus plug container comprising a 13.5 cm length of latex rubber tubing with fixed end (McMaster-Carr: part # 5234K34—Super-Soft Latex Rubber Tubing, Semi-Clear, 5/16″ inner diameter (ID), 7/16″ outer diameter (OD)). The bench model station was staffed by a data recorder and research personnel who facilitated the exchange of fluid media and handed the bronchoscopes to the clinicians according to the randomization scheme.

**Fig. 1:**
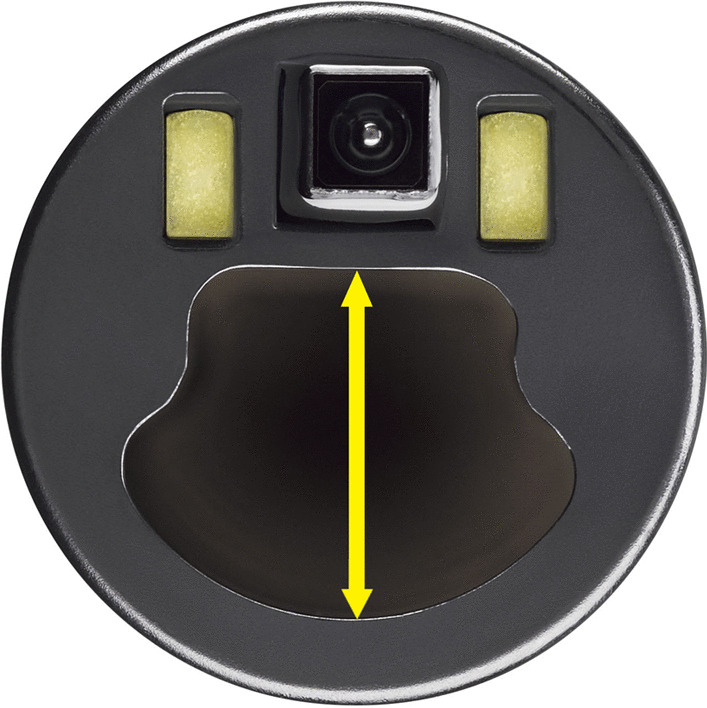
EXALT Model B single-use bronchoscope tested in the current study. Yellow arrow shows the working channel diameter

In May and June 2021, 15 physicians (Pulmonary and Critical Care, Surgical Critical Care, Anesthesiology and Thoracic Surgery specialists) at two US sites completed simulated bronchoscopy tasks according to a study schedule that used block randomization by bronchoscope diameter, then by brand, after which three bronchoscopy tasks were completed in order. The physicians included five pulmonary fellows and 10 attending physicians in pulmonary and critical care medicine (eight), thoracic surgery (one) and anesthesia (one). Their bronchoscopy experience level ranged from fellow-level (five) to attending-level for 0–5 years (one) or > 5 years (nine). Each physician performed each of the three tasks once using each of three sizes of both single-use bronchoscope models (18 tasks per physician): EXALT™ Model B (Boston Scientific Corporation, Marlborough, Massachusetts, USA), and the marketed aScope 4™ (Ambu®, Ballerup, Denmark), each in Slim (3.8 mm OD), Regular (5.0 mm OD), and Large (5.8 mm OD) sizes. Technical specifications were similar between same-sized scopes (Table 1), but the “clamshell”-shaped working channel of the EXALT Model B (Fig. 1) differs significantly from the circular-shaped working channel of the aScope 4.

**Table 1:** Technical specifications of the tested devices

Attribute	Slim		Regular		Large	
	aScope 4^a^	EXALT Model B	aScope 4^a^	EXALT Model B	aScope 4^a^	EXALT Model B
Field of view	85°	90°	85°	90°	85°	90°
Articulation angle	180° up, 180° down	180° up, 180° down	180° up, 180° down	180° up, 180° down	180° up, 160° down	180° up, 180° down
Insertion tube outer diameter (OD)	3.8 mm	3.8 mm	5.0 mm	5.0 mm	5.8 mm	5.8 mm
Working length	60 cm	60 cm	60 cm	60 cm	60 cm	60 cm
Average working channel diameter	1.2 mm	1.2 mm	2.2 mm	2.2 mm	2.8 mm	2.8 mm
Minimum working channel diameter	1.2 mm	1.0 mm	2.0 mm	2.0 mm	2.6 mm	2.6 mm
Minimum endotracheal tube size compatibility	5.0 mm	5.0 mm	6.0 mm	6.0 mm	7.0 mm	7.0 mm
Minimum double lumen tube size compatibility	35Fr	35Fr	41Fr	41Fr	N/A	N/A

^a^From: https://www.ambu.com/endoscopy/pulmonology/bronchoscopes/product/ambu-ascope-4-slim

https://www.ambu.com/endoscopy/pulmonology/bronchoscopes/product/ambu-ascope-4-regular

https://www.ambu.com/endoscopy/pulmonology/bronchoscopes/product/ambu-ascope-4-large

Accessed on October 29, 2021

**Fig. 2:**
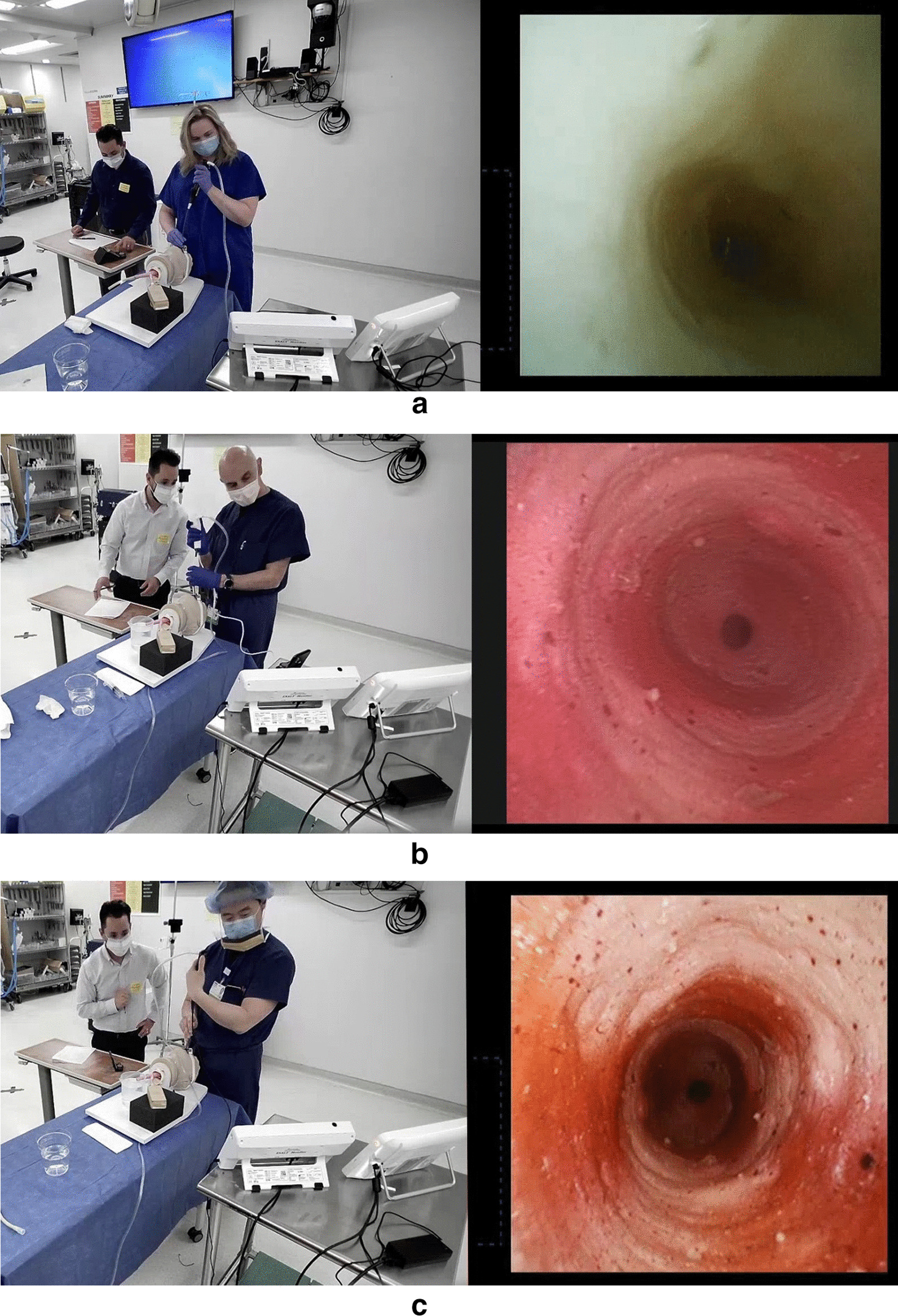
Bronchoscopy bench simulation. Investigators clear a “mucus” plug (**a**) and view “bronchi” (**b** and **c**) during bench model simulation

Wall suction was used for 14 physicians who participated in the study at Lahey Hospital (Burlington, Massachusetts, USA). A freestanding suction pump (MadaVac Aspirator, Model 172BS-II, MADA Medical Products, Inc., Carlstadt, New Jersey, USA) was used by one physician who participated in the study at a Boston Scientific Corporation laboratory (Marlborough, Massachusetts, USA). In all cases that used wall suction at the hospital, the wall valve was fully open, creating a suction pressure that was consistently approximately 360 mm Hg. This was well below the maximum recommended vacuum pressure of 85 kPa (638 mmHg) during suctioning recommended in the aScope 4 Instructions for Use[14]. To maintain consistency in pressure, the freestanding suction pump used by one physician at the corporate laboratory was also set to 360 mm Hg. For optimal visualization, the bronchoscopes were used with their dedicated monitors, namely the EXALT Model B was used with the EXALT monitor and the aScope 4 was used with the aView 2 Advance monitor. The physicians’ ability to complete the following three tasks, qualitative ratings for visualization and quantitative measurements of device performance were recorded on standard data collection forms (Fig. 2):

#### Task 1: water and visualization

Advance the bronchoscope to the carina, then the left main airway of the model, and rate visualization. Withdraw the bronchoscope back to the carina, then advance to the right main airway and into the container of water keeping the tip fully submerged with the bronchoscope in a straight configuration. As instructed by the alarm of a timer, hold the suction button down continuously for 30 s. Remove the bronchoscope from the water container.

#### Task 2: “mucus” mass suction

Advance the bronchoscope to the carina, then the right main airway and into the container of “mucus” keeping the tip fully submerged with the bronchoscope in a straight configuration. As instructed by the alarm of a timer, hold the suction button down continuously for 30 s. Remove the bronchoscope from the “mucus” container.

#### Task 3: “mucus” plug suction

Advance the bronchoscope to the carina, then the right main airways, and into the “mucus” plug container. Clear the “mucus” plug using standard technique to re-establish airway patency. If not cleared after 30 s, remove the bronchoscope to clear the channel, reinsert the bronchoscope into the right main airway, and suction for an additional 30 s. If the bronchoscope was plugged, the physician can flush out the mucus plug in the working channel using a syringe between the first and second pass, as is commonly done in the field. Remove the bronchoscope from the model.

### Endpoints

The primary endpoint was the mean amount of “mucus” mass removed during 30 s of suctioning in Task 2. Other endpoints included mean amount of water aspirated in 30 s (Task 1); completion rate and time and number of bronchoscope passes for Task 3 (clearance of “mucus” plug); and median ratings on a scale from one to 10 (best) for visualization attributes and performance for “mucus” suction. For Task 3, a maximum of 60 s and two passes were allowed for successful clearance of the “mucus” plug, and cases exceeding 60 s and/or two passes were considered failures.

### Sample size calculation

A preliminary experiment was conducted by 15 Boston Scientific Corporation personnel to estimate scope performance and to establish assumptions for the sample size calculation. Differences in magnitude of the primary endpoint measure were so large and variability so small between scopes of equivalent size (i.e. Slim vs. Slim, Regular vs. Regular, and Large vs. Large) that the study would be overpowered to compare similar-sized scopes [15]. Because the EXALT Model B Regular scope appeared to show a modest advantage over the Ambu Large scope for “mucus” suction, this comparison was used for the power calculation. In the preliminary experiment, EXALT Model B Regular suctioned a mean (${\mu }_{Model B})$ of 43 ± 4 g of “mucus.” aScope 4 Large suctioned a mean (${\mu }_{aScope 4}$) of 39 ± 5 g of “mucus.” The correlation by each participant was calculated to be about 0.4. These assumptions were the basis for the hypothesis:

$${H}_{O}: {\mu }_{aScope 4}={\mu }_{Model B}$$

$${H}_{O}: {\mu }_{aScope 4}\ne {\mu }_{Model B}$$

Using a paired t-test with a two-sided alpha of 0.05 and power of 80%, 15 pairs were required to show that the group means were different.

### Statistical analysis

Paired t-tests were used to compare the primary endpoint results. Volume suctioned and number of bronchoscope passes to complete Task 3 were expressed as means and standard deviations, with the number of passes being tested using a negative binomial model. Because visualization scores, percentage of “mucus” plug cleared and performance ratings did not follow a normal distribution, nonparametric analyses were conducted and summary statistics were presented as medians and ranges. The Kruskal–Wallis test was used to test the hypothesis that visualization score distributions were the same between types of bronchoscopes. *P* values < 0.05 were considered statistically significant. Statistical analyses were performed using SAS 9.4 software (SAS Institute Inc., Cary, NC, USA).

## Results

### Task 1: water and visualization

The mean mass of water suctioned in 30 s was significantly greater for EXALT Model B compared to aScope 4 across all scopes of equal size (126.7 ± 7.9 g vs. 55.5 ± 4.9 g for Slim, 427.8 ± 42.5 g vs. 244.8 ± 67.9 g for Regular, and 604.7 ± 62.2 g vs. 403.0 ± 33.7 g for Large respectively, *p* < 0.001 for all comparisons).

### Task 2: “mucus” mass suction

The mean masses of “mucus” suctioned by the aScope 4 Large and EXALT Model B Regular bronchoscopes were similar (41.5 g vs. 41.8 g respectively, *p* = 0.914) (Table 2, Additional file 1: Video 1). In same-sized scope comparisons, EXALT Model B aspirated a significantly greater mass of “mucus” compared to aScope 4 (4.3 g vs. 1.5 g for Slim, 41.8 g vs. 15.4 g for Regular, and 72.9 g vs. 41.5 g for Large respectively, *p* < 0.001 for all comparisons).

**Table 2:** Primary endpoint: “mucus” mass suctioned in 30 s in Task 2 (N = 15)

Comparison of aScope 4 size versus EXALT Model B size	Mean mass of “mucus” suctioned in 30 s (g)		*P* value
	aScope 4	EXALT Model B	
Large versus Regular^a^	41.5 ± 5.7	41.8 ± 8.3	0.914
Slim versus Slim	1.5 ± 0.6^b^	4.3 ± 1.3	< .001
Regular versus Regular	15.4 ± 2.9	41.8 ± 8.3	< .001
Large versus Large	41.5 ± 5.7	72.9 ± 9.0	< .001

^a^Based on a preliminary experiment, the study hypothesis was that the EXALT Model B regular scope would suction significantly more “mucus” than the aScope 4 large scope (not confirmed)

^b^There were 14 observations for aScope 4 Slim because one physician was called away for patient care and could not complete this task

### Task 3: clearance of “mucus” plug

The percentage of cases that achieved successful “mucus” plug clearance increased with increasing bronchoscope diameter, and was similar between aScope 4 and EXALT Model B scopes of equal size for all 3 device sizes (Table 3, Additional file 1: Video 1). Median Task 3 completion time was 60.0 s (maximum time allowed) for both Slim bronchoscopes, but was significantly lower for EXALT Model B compared to aScope 4 in both the Regular and Large sizes (42.0 s vs. 55.0 s, *p* = 0.002, and 23.0 s vs. 30.0 respectively, *p* = 0.005 respectively) (Table 3). The mean number of passes required to complete Task 3 ranged from 1.2 (EXALT Model B Large) to 2.0 (aScope 4 Slim; maximum 2.0 passes allowed) and was similar between models (Table 3).

**Table 3:** “Mucus” plug clearance in Task 3 (N = 15)

Task 3 measure	Bronchoscope model		*P* value
	aScope 4	EXALT Model B	
Median percentage of mucus plug cleared			
Slim	10.0 (0.0–100.0)^a^	45.0 (5.0–100.0)	0.043
Regular	80.0 (5.0–100.0)	95.0 (60.0–100.0)	0.095
Large	95.0 (50.0–100.0)	99.0 (80.0–100.0)	0.066
Mucus plug cleared			
Slim	7.1% (1/14)^a^	13.3% (2/15)	0.999
Regular	60.0% (9/15)	93.3% (14/15)	0.080
Large	93.3% (14/15)	100.0% (15/15)	0.999
Median completion time (seconds)			
Slim	60.0 (60.0–60.0)^a^	60.0 (30.0–60.0)	0.164
Regular	55.0 (30.0–60.0)	42.0 (13.0–60.0)	0.002
Large	30.0 (16.0–60.0)	23.0 (10.0–45.0)	0.005
Mean number of passes			
Slim	2.0 ± 0.0^a^	1.9 ± 0.3	0.899
Regular	1.9 ± 0.4	1.6 ± 0.5	0.584
Large	1.5 ± 0.5	1.2 ± 0.4	0.532

^a^14 observations for aScope 4 Slim after one physician was called away for patient care and could not complete task

### Visualization

Median visualization ratings were high overall, ranging from 7.0 to 10.0 (Table 4). The EXALT Model B using the EXALT Monitor was rated significantly higher than aScope 4 using aView 2 Advance tablet in all visualization attributes (*p* ranging from 0.001 to 0.029).

**Table 4:** Visualization ratings (N = 15)

Attribute	Median rating (range)		*P* value
	aScope 4 / aView 2 Advance monitor	EXALT model B/EXALT monitor	
Definition	8.0 (7.0−10.0)	9.0 (8.0−10.0)	0.001
Color	8.0 (7.0−10.0)	9.0 (7.0−10.0)	0.009
White balance	8.0 (6.0−10.0)	9.0 (7.0−10.0)	0.029
Far field resolution	7.0 (6.0−10.0)	9.0 (7.0−10.0)	0.001
Near field resolution	9.0 (7.0−10.0)	10.0 (8.0−10.0)	0.018

### Qualitative performance ratings for “mucus” suction

Ratings for ease of use and operator comfort ranged from 9.0 to 10.0, with no significant difference between same-sized bronchoscope models (Table 5). Ratings for perception of efficacy were significantly lower for aScope 4 compared to EXALT Model B in the Slim and Regular sizes (1.0 vs. 2.0, *p* = 0.045 and 5.0 vs. 8.0 respectively, *p* < 0.001) and similar for the Large bronchoscope models (7.0 for aScope 4 vs. 9.0 for EXALT Model B, *p* = 0.237) (Table 5).

**Table 5:** Median performance ratings for “mucus” suction

Peformance characteristic	Median rating (range)^a^		*P* value
	aScope 4	Model B	
Ease of use			
Slim	10.0 (7.0−10.0)^b^	10.0 (6.0−10.0)	0.530
Regular	10.0 (8.0−10.0)	10.0 (8.0−10.0)	0.562
Large	9.0 (7.0−10.0)	10.0 (8.0−10.0)	0.195
Perception of efficacy			
Slim	1.0 (1.0−9.0)^b^	2.0 (1.0−7.0)	0.045
Regular	5.0 (3.0−8.0)	8.0 (5.0−10.0)	< .001
Large	7.0 (3.0−10.0)	9.0 (4.0−10.0)	0.237
Operator comfort			
Slim	9.5 (7.0−10.0)^b^	10.0 (8.0−10.0)	0.425
Regular	10.0 (7.0−10.0)	10.0 (8.0−10.0)	0.600
Large	9.0 (6.0−10.0)	10.0 (6.0−10.0)	0.469

^a^Rated on a scale of one (worst) to 10 (best)

^b^14 observations for aScope 4 Slim after one physician was called away for patient care and could not complete task

## Discussion

This is the first study of the technical performance of a new single-use flexible bronchoscopy. Procedural experts from several disciplines conducted a bench model simulation of bronchoscopy tasks using the new single-use bronchoscope compared to a marketed 4^th^-generation single-use bronchoscope in three sizes each. The regular size of the new bronchoscope aspirated a similar amount of “mucus” compared to the large size of the marketed bronchoscope. For all same-sized comparisons, a significantly greater mass of “mucus” was aspirated by the new device. Subjective ratings for performance were similar or better, and for all categories of visualization were better, for the new device versus the marketed comparator.

Flexible bronchoscopy was introduced into clinical practice in 1966 and has become the most frequently performed standard invasive procedure in pulmonary medicine [16]. Although rigid bronchoscopy may be indicated for massive hemoptysis or other specific therapeutic circumstances [4], flexible bronchoscopy has replaced rigid bronchoscopy for many diagnostic and therapeutic uses in patients without contraindications (uncorrectable hypoxemia, uncontrolled arrhythmias, lack of proper equipment and skilled personnel) [16]. The portability, immediate availability, and theoretical reduced risk of clinically relevant infections confer an advantage of using single-use over reusable flexible bronchoscopes in certain scenarios in the bronchoscopy and intensive care units [17]. However, because studies have not been performed comparing single-use versus reusable bronchoscopes in complex bronchoscopic procedures, a 2022 systematic review recommended that use of single-use bronchoscopes should be limited to flexible bronchoscopic intubation, simple therapeutic aspirations, BAL, and in low-risk percutaneous tracheostomy procedures until further evidence for more widespread use becomes available [17].

The new single-use bronchoscope in the current study suctioned a significantly larger amount of water and “mucus” and more efficiently cleared “mucus” plug material compared to marketed same-diameter comparators. Because the suction pressure setting was the same for both brands of devices, the better performance of the new device might be associated with the “clamshell” shape of the working channel (Fig. 1) that increases the fractional area of the working channel for bronchoscopes with the same outer diameter in the new single-use bronchoscope compared to the marketed device. The equivalent “mucus” suction performance of the EXALT Model B Regular (5.0 mm OD) and aScope 4 Large (5.8 mm OD) sizes could improve management of retained secretions when a high degree of suction efficacy is needed to establish patency within an occluded airway or when a smaller size endotracheal tube is in place in patients with poor pulmonary reserve to decrease interruption in ventilation. Although mass and proportion of “mucus” suction were significantly greater for the slim version of the new single-use bronchoscope, both slim models had low overall “mucus” suction performance and low perceived efficacy. This suggests size-induced performance limits on any slim bronchoscope used through pediatric or double-lumen smaller adult endotracheal tubes. Nevertheless, the improved efficacy of mucus suctioning with the new slim model bronchoscope (Table 2) may prove useful in scenarios where larger diameter bronchoscopes cannot be employed, such as during lung operations with single lung ventilation using a narrow lumen double-lumen endotracheal tube.

Single-use bronchoscopes are not to be stored and reused, even in the same patient, because of the same risk of cross-contamination seen with repeated use of conventional bronchoscopes [18]. Impact on the environment from the greater volume of single-use endoscopes used must be considered. Single-use bronchoscopes have been marketed for years, but recycling programs pose logistical and financial challenges and are not known to be adopted [19]. Similarly, use of cleaning materials and personal protective equipment required with reusable bronchoscopes might have comparable or potentially higher material and energy consumption as well as emissions of CO_2_ equivalents compared with single-use flexible bronchoscopes [7]. Environmental conservation efforts must continue as single-use and reusable endoscope technology develops.

The current study has several strengths and limitations. Strengths include protocol-guided testing of the new bronchoscope and comparator on a fixed anatomical model. The study used block randomization and all data were collected except for one incomplete set of tasks by one physician. Limitations include the small study size and potential bias from an unblinded comparison because of known physical features and appearance (e.g. white vs. black color) of the marketed bronchoscopes. We used a synthetic bench model with siliconized rubber “airways”; these “airways” lacked lobar or segmental branches and would be less likely to collapse than human airways in response to the suction pressures used in the study. We cannot conclude that results using this model would be similar to results in human airways. However, bronchoscopies performed in the intensive care unit often treat mucus plugging of the mainstem bronchi that impacts oxygenation and ventilation, and the study simulated the required bronchoscopic intervention for removal of mucus plugs from these larger airways to improve the respiratory status of the patient. Wall suction pressure used during the study was higher than that recommended for procedures such as BAL [20–22]. Different results might have been obtained in clinical practice where lower suction pressure is used. Further study of performance of the tested devices at clinically relevant pressure-flow settings is warranted. The superiority hypothesis for “mucus” suctioned by EXALT Model B Regular compared to aScope 4 Large was not confirmed (performance similar). However, EXALT Model B suctioned significantly more “mucus” than aScope 4 in all same-sized device comparisons. Finally, this preclinical study utilized an airway simulation model and caution should be exercised in extrapolating these results to human airways; clinical studies are needed to confirm the results from this study.

## Conclusions

A regular-sized first-generation single-use bronchoscope aspirated “mucus” as well as a large marketed comparator in a bench simulation of bronchoscopy tasks. For all same-sized scope comparisons, volume of mucus suctioned in 30 s was superior for the new single-use bronchoscope. Visualization was rated more highly, and general performance was rated similar for the new device compared to the marketed comparator. This small bench study’s positive findings should be confirmed by clinical studies.

## Supplementary Information

**Additional file 1: Video 1**. Physicians perform three simulated bronchoscopy tasks using three sizes of two single-use bronchoscopes in a bench model.

## Data Availability

The data analytic methods and study materials for this study may be made available to other researchers in accordance with the Boston Scientific Data Sharing Policy (http://www.bostonscientific.com/en-US/data-sharing-requests.html).

## Abbreviations

BAL: Bronchoalveolar lavage
CO_2_: Carbon dioxide
Fr: French
ICU: Intensive care unit
ID: Inner diameter
kPa: Kilopascal
OD: Outer diameter
SAS: Statistical Analysis Software
